# Targeting Oxidative Stress and Inflammation to Prevent Ischemia-Reperfusion Injury

**DOI:** 10.3389/fnmol.2020.00028

**Published:** 2020-03-05

**Authors:** Liquan Wu, Xiaoxing Xiong, Xiaomin Wu, Yingze Ye, Zhihong Jian, Zeng Zhi, Lijuan Gu

**Affiliations:** ^1^Department of Neurosurgery, Renmin Hospital of Wuhan University, Wuhan, China; ^2^Central Laboratory, Renmin Hospital of Wuhan University, Wuhan, China; ^3^Department of Anesthesiology, Zhejiang Provincial People’s Hospital, People’s Hospital of Hangzhou Medical College, Hangzhou, China; ^4^Department of Pathology, Renmin Hospital of Wuhan University, Wuhan, China

**Keywords:** oxidative stress, inflammation, cerebral ischemia/reperfusion injury, neuroprotective, signaling pathways

## Abstract

The cerebral ischemia injury can result in neuronal death and/or functional impairment, which leads to further damage and dysfunction after recovery of blood supply. Cerebral ischemia/reperfusion injury (CIRI) often causes irreversible brain damage and neuronal injury and death, which involves many complex pathological processes including oxidative stress, amino acid toxicity, the release of endogenous substances, inflammation and apoptosis. Oxidative stress and inflammation are interactive and play critical roles in ischemia/reperfusion injury in the brain. Oxidative stress is important in the pathological process of ischemic stroke and is critical for the cascade development of ischemic injury. Oxidative stress is caused by reactive oxygen species (ROS) during cerebral ischemia and is more likely to lead to cell death and ultimately brain death after reperfusion. During reperfusion especially, superoxide anion free radicals, hydroxyl free radicals, and nitric oxide (NO) are produced, which can cause lipid peroxidation, inflammation and cell apoptosis. Inflammation alters the balance between pro-inflammatory and anti-inflammatory factors in cerebral ischemic injury. Inflammatory factors can therefore stimulate or exacerbate inflammation and aggravate ischemic injury. Neuroprotective therapies for various stages of the cerebral ischemia cascade response have received widespread attention. At present, neuroprotective drugs mainly include free radical scavengers, anti-inflammatory agents, and anti-apoptotic agents. However, the molecular mechanisms of the interaction between oxidative stress and inflammation, and their interplay with different types of programmed cell death in ischemia/reperfusion injury are unclear. The development of a suitable method for combination therapy has become a hot topic.

## Introduction

**Figure 1:**
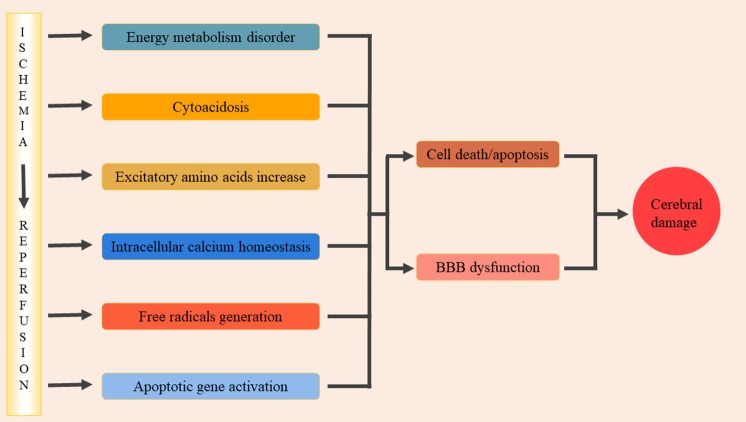
The pathophysiological mechanisms of cerebral ischemia/reperfusion injury. The main pathophysiological mechanisms include: energy metabolism disorders, cellular acidosis, synthesis or release of excitotoxic amino acids into doubling, intracellular calcium homeostasis, free radical production, activation of apoptotic genes. These factors interacted and formed a complex regulatory network, and then lead to a series of pathological cascade reactions. It lead directly or indirectly nerve cells apoptosis/death, damage the blood-brain barrier and cause brain edema, and eventually lead to neural dysfunction.

Cerebral ischemia-reperfusion injury (CIRI) is a complex pathophysiological process that can cause severe damage to brain functioning. Significant progress has been made in understanding the pathophysiological mechanisms of cerebral ischemia-reperfusion injury and, at present, the main mechanisms include: energy metabolism disorders, cellular acidosis, synthesis or release of excitotoxic amino acids into doubling, intracellular calcium homeostasis, free radical production, and activation of apoptotic genes (Lin et al., 2016; Figure 1). These various factors interact to form a complex regulatory network, resulting in a series of pathological cascades that directly or indirectly cause apoptosis/death of nerve cells, disrupt the blood-brain barrier resulting in brain edema, and ultimately lead to neurological deficits (Xiong et al., 2018). During cerebral ischemia-reperfusion, oxidative stress occurs and a large number of inflammatory cytokines are present in the center and periphery of the ischemic focus. The activation and infiltration of inflammatory cells and the synthesis and secretion of adhesion molecules are cascade reactions that enhance and promote one another. Through certain oxidative stress and inflammatory signaling pathways, brain tissue may suffer from ischemic and oxidative stress-inflammatory injuries. Therefore, oxidative stress-inflammation plays an important role in CIRI.

## The Role of Oxidative Stress in CIRI

Oxidative stress refers to the pathological process of tissue damage caused by the excessive production, or reduced scavenging capacity, of reactive oxygen species (ROS) during metabolic processes, resulting in an imbalance between oxidative and antioxidant systems. The development of CIRI is complex, but oxidative stress and free radical chain reactions are the core pathological aspects. Mitochondrial dysfunction and oxidative stress injury caused by energy metabolism failure are two of the main factors that aggravate the irreversible damage caused by cerebral ischemia/reperfusion. When mitochondrial dysfunction and oxidative stress damage the oxidation/antioxidation balance in the body, neuronal mitochondria produce large amounts of ROS and hydroxyl radicals, leading to neuron death (Wu M. Y. et al., 2018). Actively protecting mitochondrial function, anti-oxidation, free radical scavenging, and slowing down oxidative stress have become effective strategies in saving neurons from the pathological processes of cerebral ischemia reperfusion.

### ROS

ROS include superoxide radicals, lipid radicals, and hydroxyl radicals. ROS can be involved as important signaling molecules in cell signal transduction, thus maintaining the normal function of cells. Cerebral ischemia/reperfusion leads to oxidative phosphorylation uncoupling, thereby increasing ROS production and lipid peroxidation.

Excessive ROS react with surrounding biological macromolecules such as lipids, proteins, sugar and DNA through a series of cascade reactions, which affects cell function and even causes cell death. The toxic mechanism of ROS mainly includes: (1) acting on polyunsaturated fatty acids causes lipid peroxidation in mitochondrial membrane (Mukherjee et al., 2019); (2) inducing cross-linking of macromolecules such as DNA, RNA, polysaccharides and amino acids, then the cross-linked macromolecules lose their original activity or function (Li S. et al., 2019); (3) damaging endothelial cells and causing an increased permeability in blood-brain barrier due to their high reactivity to oxidized lipids and proteins (Wang Y. et al., 2019); (4) mediating inflammation and immune response by stimulating the expression of cytokines and adhesion molecules, leading to and aggravating brain tissue reperfusion injury (Xu X. et al., 2018); (5) promoting polymerization and degradation of polysaccharide molecules. Free radicals can attack a wide range of nerve membranes and blood vessels that are rich in unsaturated fatty acids, triggering lipid peroxidation cascade effect, protein denaturation, polynucleotide strand breaks, base re-modification, cell structure integrity destruction, and thus the permeability, ion transport, and barrier functions of cell membrane are all seriously affected (Xing et al., 2018); and (6) leading to increased release of excitatory amino acid (EAA) that promotes the development of delayed neuron death after cerebral ischemia (Zhang et al., 2015a).

### The Role of Anti-oxidative Stress Substances in CIRI

#### Enzymatic and Non-enzymatic Antioxidant Systems

The body contains an endogenous anti-oxidation system that ensures that the generation and elimination of free radicals are in a dynamic equilibrium, protecting the structure and function of cells. Total antioxidant capacity (T-AOC) is an important index reflecting the body’s ability to compensate for excess free radicals *via* the antioxidant system, the state of free radical metabolism in the body, and the body’s antioxidant function. Higher serum T-AOC levels are associated with mortality in patients with severe ischemic stroke and could be used as a prognostic biomarker (Lorente et al., 2016). T-AOC includes both enzymatic and non-enzymatic systems. The enzymatic systems include superoxide dismutase (SOD), thioredoxin (Trx), paraoxonase (PON), glutathione peroxidase (GSHPx), catalase (CAT), glutathione s-transferase (GST), and others. Non-enzymatic systems include glutathione (GSH), vitamin A, vitamin C, Vitamin E, and carotenoids (Kryl’skii et al., 2019). Studies have confirmed that T-AOC regulation can protect neuronal damage in CIRI (Deng et al., 2015; Lin et al., 2015; Kryl’skii et al., 2019). α-lipoic acid exerted its neuroprotective effects through reversing the levels of oxidative parameters, including malondialdehyde (MDA), nitric oxide (NO), T-AOC, and SOD to their normal state in rat brains following CIRI (Deng et al., 2015). Lin et al. found that neuronal damage in the hippocampal CA1 area was significantly reduced after cerebral ischemia/reperfusion in SOD transgenic mice (Lin et al., 2015; Xu X. et al., 2018).

#### Nitric Oxide Synthase (NOS)

**Figure 2:**
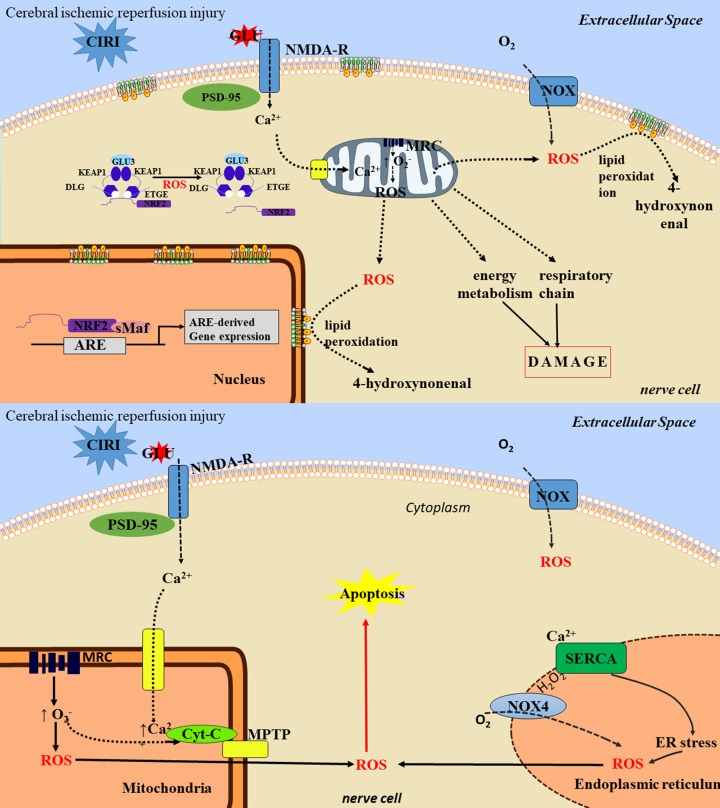
The toxic mechanisms of cerebral ischemia/reperfusion injury. The toxic mechanisms of reactive oxygen species (ROS) in ischemia/reperfusion injury are mainly as follows: (1) nerve cell necrosis: ROS can lead to protein degeneration and enzyme inactivation, which can lead to damage of mitochondrial respiratory chain, energy generation barrier and cell death. ROS reacts with cell membrane lipids to produce lipids peroxide. After lipid peroxide degradation, the toxic products such as 4-hydroxyl can be formed, thus damaging neurons and causing neuronal cell necrosis. (2) neuronal apoptosis: ROS can induce neuronal apoptosis through activation of multiple pathways, such as mitochondrial pathway and endoplasmic reticulum pathway, and involves multiple signaling pathways. ① the mitochondria pathway: ROS attacks mitochondria to cause the cytochrome C oxidase phosphorylation, mitochondrial transmembrane potential rising, MPTP remains open and release. Through activation of apoptotic protease activating factor 1, apoptosis inducing factor, apoptotic protease activating factor, the Bcl-1 family proteins, ROS starts the cascade activation of apoptosis pathway to promote cell apoptosis, ② the endoplasmic reticulum stress (ERS) pathway: the ERS refers to lack of oxygen, glucose, or lack of nutrients or lipids, virus infection, toxin and excessive load drug harmful factors such as steady and smooth endoplasmic reticulum, Ca^2+^ balance disorders and non-folded protein misfolding then gathered in the endoplasmic reticulum. ERS can activate the apoptotic signaling pathway, and eventually induce apoptosis, thereby aggravating cerebral ischemia/reperfusion injury (CIRI).

Nitric oxide synthase (NOS) catalyzes L-arginine and molecular oxygen to produce nitric oxide (NO), which has two effects in ischemic injury, neurotransmission, and neurotoxicity. There are three types of NOS: endothelial NOS (eNOS), neuronal NOS (nNOS), and inducible NOS (iNOS; Pradhan et al., 2018). In the early stages of cerebral infarction, NO production is promoted by eNOS synthesis. Although eNOS accounts for only 10% of total NO, it plays an important role in promoting vasodilation, increasing cerebral blood flow, and protecting neurons from damage. However, in the late stage of cerebral ischemia, NO is produced by iNOS and nNOS, which exacerbates neurotoxicity and causes delayed neuronal injury. It has been demonstrated that honokiol can reduce nNOS-derived NO by decreasing the membrane translocation of nNOS, thus improving cerebral infarction and edema after ischemia (Hu et al., 2013). Some drugs can upregulate the expression or activity of eNOS, increase cerebral blood flow, and protect neurons from cerebral ischemic injury (Watanabe et al., 2016; Mahmood et al., 2017). Mitogen- and stress-activated protein kinase (MSK) exerts a protective effect on rats with focal ischemia-reperfusion injury through its anti-apoptotic effect on neurons and anti-inflammatory effect on astrocytes by decreasing the expression of inducible nitric oxide synthase (iNOS) and increasing the expression of interleukin-10 (IL-10; Esmaeilizadeh et al., 2015). Vitamin E and crocin can reduce oxidative stress damage during ischemia/reperfusion by regulating eNOS and iNOS expression (specifically, by reducing iNOS and increasing eNOS; Zhu et al., 2018; Figure 2).

#### Anti-oxidative Stress Signal Pathway

##### Nrf2/ARE Signaling Pathway

Nrf2/ARE is an important endogenous anti-oxidative stress signaling pathway. Nrf2 belongs to the alkaline leucine zipper family, is the most active transcription factor regulating antioxidant responses, and is a receptor for oxidative stress (Hu et al., 2015; Chen X. et al., 2016). Keap1 is its specific receptor. Activation of the Nrf2/ARE signaling pathway can regulate cellular anti-oxidative stress and protect against cerebral ischemia/reperfusion. In the resting state, Nrf2 is coupled with Keap1 in the cytoplasm, so that Nrf2 activity is relatively inhibited. However, Nrf2 dissociates from Keap1 in two situations: (1) Keap1 configuration changes when attacked by electrophilic substances or ROS, and (2) through the protein kinase C (PKC) pathway. In the latter, phosphorylation of Nrf2 leads to the uncoupling of Nrf2 and Keap1, and Nrf2 then undergoes nuclear translocation, binds to musculoaponeurotic fibrosarcoma protein (Maf) to form a heterodimer, and then binds ARE. This initiates ARE-regulated target gene expression, including anti-inflammatory proteins, antioxidant enzymes, and growth factors, among others (Itoh et al., 2015). ARE regulates the expression of many antioxidant genes, including hemeoxygenase-1 (HO-1), quinine oxidoreductase 1 (NQO-1), and GST (Alfieri et al., 2013). These enzymes can resist oxidative stress and damage produced by various internal and external stimuli, and promote the return to equilibrium of oxygen free radicals. Certain drugs and naturally extracted compounds (sulforaphane, ginkgo extract, white squash, andrographolide derivative CX-10, rutaecarpine, ginsenoside Rg1, and levocarnitine, among others) are able to protect the body from stroke damage by activating the Nrf2 signaling pathway (Alfieri et al., 2013; Chu et al., 2019; Han et al., 2019; Liu and Zhang, 2019; Yang et al., 2019). *In vitro*, activated Nrf2 has strong neuroprotective properties and can inhibit stroke injury mechanisms such as glutamate toxicity, H_2_O_2_ exposure, and Ca^2+^ overloading (Xu P. et al., 2018). Furthermore, Nrf2 inducers are able to reverse free radical-induced neuronal cell death (Han et al., 2019). When the Nrf2 inducer tBHQ is injected into cerebral ischemia/reperfusion rat and mouse models, sensorimotor and histological functions improve (Hou et al., 2018). Nrf2-knockout mice are more susceptible to ischemic brain damage and neurological impairment than wild type mice (Zhang et al., 2017) Current studies indicate that the signaling pathways involved in Nrf2 nuclear translocation regulation include MAPKs, PKC, and PI3K/Akt.

Kitagawa et al. (1999) first demonstrated that the PI3K/Akt signaling pathway was involved in protection from CIRI in a middle cerebral artery occlusion (MCAO) rat model (Kitagawa et al., 1999). The PI3K/Akt signaling pathway induces Nrf2 nuclear translocation by modulating the recombination of motor proteins, thereby facilitating the production of downstream anti-oxidative stress substances (Xu Y. P. et al., 2017). *In vitro*, shikonin can induce the expression of Nrf2 and HO-1 by regulating the PI3K/Akt signaling pathway (Huang et al., 2015). The application of the PI3K specific blocker LY-294002 can inhibit Nrf2 nuclear translocation (Zheng et al., 2014). ERK/MAPK is involved in cell growth, proliferation, differentiation, transformation, and other protective mechanisms during ischemia/reperfusion and inhibits apoptosis, while P38 and c-Jun are involved in the development of neuronal apoptosis. Chemical inducers can activate the ERK signaling pathway to promote the expression of downstream target genes of ARE, which may be related to the direct phosphorylation of Nrf2 by ERK and may promote Nrf2 nuclear translocation (Chang et al., 2015; Cen et al., 2019). PKC is a family of phospholipid-dependent serine/threonine kinases, each subtype of which participates in the regulation of apoptosis, the inflammatory response, oxidative stress, and other pathophysiological processes. Isozymes of PKC family, specifically PKCγ and PKCε are involved in oxygen-glucose deprivation (OGD) induced intracellular responses, which leads to neuronal death. Iisozyme-specific modulation of PKC activity may serve as a promising therapeutic route for the treatment of acute cerebral ischemic injury (Surendran, 2019). Activation of epsilon protein kinase c (PKCε) during cerebral ischemic preconditioning (CIP) can increase mitochondrial membrane potential, decrease ROS production, and increase phosphorylation of mitochondrial K^+^ ATP channels (Thompson et al., 2012). Protocatechualdehyde (PCA), a major bioactive and effective ingredient extracted from Chinese traditional medicines, such as the roots of *Salvia miltiorrhiza*, leaves of *Stenoloma chusanum* (L.) Ching and Ilex chinensis Sims, has been proven to have putative antioxidant activities (Guo et al., 2017). PCA significantly increased the Nrf2 and HO-1 expressions in the MCAO ischemic cerebral cortex, moreover, knockdown of PKCε also blocked PCA-induced Nfr2 nuclear translocation, HO-1 expression, and neuroprotection (Guo et al., 2017). PKCε are also involved in NF-κB, JNK, P38, MAPK, adenosine receptor, and other signaling pathways (Morris-Blanco et al., 2016; Guo et al., 2017). This indicates that activated PKCε participates in various signaling pathways to protect neurons.

##### SIRT/FOXO Signaling Pathway

Sirtuins (SIRT1–7) are deacetylases that use NAD as a coenzyme and have a catalytic core region consisting of 275 amino acids. They are involved in various cellular physiological processes, such as energy metabolism, oxidative stress, and apoptosis. Their ability to resist oxidative stress has been widely studied in cerebral ischemia-reperfusion (Hou et al., 2010; Rothgiesser et al., 2010; Morris et al., 2011; She et al., 2018; Wang et al., 2018; Wu D. et al., 2018; Chang et al., 2019; Duan et al., 2019; Fusco et al., 2019; Rao et al., 2019; Xian et al., 2019; Zhao B. et al., 2019). SIRT1 activates the FOXO family, and FOXO3a has the ability to resist anti-oxidative stress and enhances ROS scavenging activity (Hou et al., 2010). SIRT1 also activates the peroxisome proliferator-activated receptor γ coactivator-1PGC1α (a transcriptional coactivator of oxidative stress) and exerts an antioxidant effect (Morris et al., 2011). By targeting SIRT1 or regulating the SIRT1 related pathway, neurons will be protected from CIRI-induced mitochondrial oxidative stress and inflammatory response (Duan et al., 2019; Fusco et al., 2019; Rao et al., 2019; Xian et al., 2019). Inflammatory cytokines can enhance the activity of SIRT2 in cells, enhance its deacetylation of NF-κB, reduce the transcriptional activity of NF-κB, and reduce the expression of inflammatory mediators, such as interleukin-6 (IL-6), matrix metalloproteinase 9 (MMP9), and cyclooxygenase-2 (COX-2), to exert antioxidant and anti-inflammatory effects (Rothgiesser et al., 2010). Further studies have also shown that SIRT2-specific activity inhibitor can aggravate neurological impairment after CIRI, and identified the downregulation of AKT/FOXO3a and p38/MAPK pathways as intermediary mechanisms that may contribute to the reduction in apoptotic cell death caused by SIRT2 inhibition (She et al., 2018; Wu D. et al., 2018). SIRT3 enhances SOD2 activity by deacetylating its lysine residues, reducing ROS levels in cells or tissues, increasing the organism’s ability to resist oxidative stress, and inhibiting apoptosis (Wang et al., 2018). Trans sodium crocetinate (TSC), the major bioactive and effective ingredient extracted from carotenoid crocetin, has been reported to exert protective effect against cerebral ischemia/reperfusion (I/R) injury (Chang et al., 2019). TSC alleviates CIRI-induced myocardial oxidative stress and apoptosis *via* the SIRT3/FOXO3a/SOD2 signaling pathway (Chang et al., 2019). In addition, Genipin, an aglycone derived from geniposide (an iridoid glycoside) that purified from the fruit of Gardenia jasminoides, also exerts a protective effect against cerebral ischemia/reperfusion (I/R) injury (Zhao B. et al., 2019). Genipin protects against CIRI by regulating the UCP2-SIRT3 signaling pathway that is involved in the CIRI as a bridge between energy metabolism and oxidative stress (Zhao B. et al., 2019). T-LAK-cell-originated protein kinase (TOPK) is involved in many biological functions, including the inhibition of apoptosis, promotion of cell growth, and anti-oxidation. TOPK has the ability to regulate oxidative stress: in H_2_O_2_-induced oxidative stress, TOPK is significantly activated, and p-TOPK levels begin to increase at 15 min, peak at 30–60 min, and return to basal levels at 120 min, while a TOPK inhibitor aggravates H_2_O_2_-induced oxidative stress damage (Zhao et al., 2014b). CIP promotes TOPK phosphorylation, significantly enhances TOPK activity, and reduces reperfusion injury (Zhao et al., 2014a). Further findings suggest that TOPK positively regulates microglia/macrophage M2 polarization by inhibiting histone deacetylase (HDAC) 1/HDAC2 activity, which regulated inflammatory responses by participating in the microglia/macrophages M1/M2 polarization (Wang et al., 2015; Zhao et al., 2016; Han et al., 2018), and may contribute to its neuroprotective effects against CIRI (Han et al., 2018). Previous mechanism studies have shown that ERK is the downstream signal of TOPK, and that the PI3K/PTEN/Akt and PBK signaling pathways are also involved in the TOPK regulation process (Gao et al., 2016; Sun et al., 2016).

## Antioxidant Therapy in CIRI

The production and release of oxygen free radicals is a key step in neuronal death. Therefore, scavenging oxygen free radicals is an important approach for the treatment of CIRI. In theory, blocking pathways or targeting the key molecules of ROS generation can reduce the oxidative damage caused by CIRI. Some progress has been made in antioxidant treatments for CIRI (Kim et al., 2009; Hu et al., 2013; Watanabe et al., 2016; Mahmood et al., 2017; Lou et al., 2018; Song et al., 2018; Xing et al., 2018; Zhu et al., 2018; Chu et al., 2019; Han et al., 2019; Li S. et al., 2019; Liu and Zhang, 2019).

### Natural Antioxidants

There are many endogenous antioxidant compounds in the human body, such as the free radical metabolic enzymes superoxide dismutase (SOD), catalase (CAT) and glutathione peroxidase, and antioxidant molecules such as vitamin E and vitamin C. Cells themselves synthesize parts of antioxidants such as glutathione. In addition to the direct action of antioxidants, indirect antioxidants such as metal complexing agents can remove free radicals generated in the body and maintain the body’s dynamic balance.

There are also many anti-oxidant ingredients naturally-occurring in plants. Arabian-type polysaccharide (GBW) extracted from *Ginkgo biloba*, polyphenols from a variety of foods, and *Sophora alopecuroides* extract oxidized alkaloids all have antioxidant effects on cerebral ischemia in animals (Alfieri et al., 2013; Yang et al., 2013, 2019; Chu et al., 2019; Han et al., 2019). In a mouse model of CIRI, it was demonstrated that a purpura extract can significantly reduce infarct size, restore the activities of antioxidants such as SOD and GSH, and decrease pro-oxidase enzyme activity by xanthine oxidase (Bora and Sharma, 2011). The protective effect of resveratrol on CIRI in mice is also related to its antioxidant properties (Dou et al., 2019). Flavonoids, presented broadly in plants and diets, have also been shown the effect and function on CIRI and cognition in experimental studies on their different flavonoid species, such as quercetine and catechin (Calis et al., 2019).

### Synthetic Antioxidant

In addition to natural extracts, studies have found that commonly used cardiovascular drugs such as simvastatin (transfusion of lipids) and trimetazidine (anti-angina drugs) have anti-CIRI effects, and that the mechanism involves anti-oxidative activity (Dhote and Balaraman, 2008; Beretta et al., 2011; Anthony Jalin et al., 2015). Lipophilic simvastatin can significantly improve the antioxidant capacity of ischemic brain tissue, while hydrophilic simvastatin has no obvious brain protection properties (Beretta et al., 2011), suggesting that increasing blood-cerebrospinal fluid barrier permeability could be an effective way to improve the treatment of CIRI with existing drugs. Edaravone, which is widely used in Asian countries, can improve mitochondrial edema, increase the expression of eNOS, reduce free radical formation, reduce cerebral infarction area, and prevent delayed neuronal damage (Fujiwara et al., 2016). It also has anti-apoptosis, anti-cell necrosis, and anti-inflammatory effects (Fujiwara et al., 2016; Song et al., 2018). NAPDH oxidase is a specific ROS-producing enzyme, particularly of NOX4, which is mainly expressed in neurons. Drugs that inhibit NOX4 may have protective effects for brain tissue after ischemia/reperfusion injury (Lou et al., 2018). Casein kinase 2 (CK2) is an important regulator of NADPH oxidase, and inactivation of CK2 in brain tissue can increase NOX2 activity, and increase ROS production and neuronal death (Kim et al., 2009). Tetramethylpyrazine analog Z-11 plays a protective role in CIRI, and the mechanisms are associated with enhancing oxidant defense systems *via* the activation of Nrf2/HO-1 and Rac-1/NADPH oxidase pathways (Li Z. et al., 2019).

## The Role of Inflammation in CIRI

Inflammatory reactions are an important pathophysiological mechanism of ischemic brain injury, participating in the occurrence and development of ischemic stroke. The expression of inflammatory cytokines in ischemic brain tissue increases significantly after cerebral ischemia/reperfusion, and this remarkable manifestation triggers various mechanisms that lead to brain tissue damage. These mechanisms include changes in vasomotor contraction, obstruction of microvessels, release of secreted cytotoxic enzymes, and generation of oxygen free radicals (Talma et al., 2016). Inflammatory cytokines not only cause nerve damage and have neurotoxic effects, but they also have certain neuroprotective effects, such as when various inflammatory cytokines interact with each other to participate in the pathological process of ischemic stroke.

### Anti-inflammatory Cytokines

#### TGF-β

TGF-β is rarely expressed in normal brain cells, and its expression is significantly increased during ischemia and after reperfusion. It can inhibit the inflammatory response of the central nervous system in early ischemia, reduce cerebral edema and infarct size, promote microvascular proliferation, and play an important role in the repair of brain tissue damage (Wang et al., 2016). TGF-β antagonists were injected into the cerebral cortex of rats with focal cerebral ischemia, and infarct volume was significantly enlarged in mice with cerebral ischemia. After hypoxic injury, administration of exogenous TGF-β can effectively reduce infarct volume. Therefore, increased expression of TGF-β in neurons can be considered a marker of neuronal survival (Bonaventura et al., 2016).

#### IL-10

IL-10 is a multifunctional cytokine that inhibits the production of inflammatory molecules and chemokines, prevents the inflammatory cascade, and reduces the effects of inflammatory damage. The possible mechanism of the protective effect of IL-10 on cerebral ischemia is its inhibition of cytokines and chemokines production at the transcriptional level (Garcia et al., 2017), and its upregulation of the expression of cytokine antagonists such as IL-1RA and soluble p55 and p75 TNFR genes *in vivo*, thereby antagonizing the pro-inflammatory effects of IL-1 and tumor necrosis factor (TNF). IL-10 also inhibits NF-κB, Ras, and other signal transduction pathways, thereby inhibiting the production of a variety of related inflammatory mediators to reduce inflammation after ischemia (Liang et al., 2015).

#### IL-13

IL-13 is produced by activated Th2 cells and inhibits the secretion of inflammatory cytokines and chemokines by mononuclear macrophages such as IL-1, IL-6, IL-8, Macrophage inflammatory protein-1, and TNF-α. It can also inhibit the antibody-dependent cell-mediated cytotoxicity of human monocytes induced by interferon-γ (IFN-γ) or IL-10. Animal experiments have shown that topical application of IL-13 can inhibit the production of inflammatory cytokines and chemokines to prevent the inflammatory cascade (Hamzei Taj et al., 2018).

#### Granulocyte Colony Stimulating Factor (G-CSF)

G-CSF is a powerful mobilizing agent for bone marrow hematopoietic stem cells, and has the ability to simultaneously mobilize hematopoietic stem cells and mesenchymal stem cells. G-CSF transforms mesenchymal stem cells into directional stem cells/neural stem cells in brain tissue, thereby repairing ischemic injury and improving neurological deficit (Balseanu et al., 2014). G-CSF can also protect ischemic neurons through G-CSF receptors on nerve cell membranes (Hamzei Taj et al., 2018). In addition, G-CSF can effectively cross the blood-brain barrier and stimulate the expression of G-CSF in the central nervous system (Tang et al., 2018). After binding to its receptor, it activates the JAK3 and STAT3 pathways to increase STAT3 levels in neurons in the peripheral zone of the ischemic foci, and STAT3 then indirectly exerts an anti-apoptotic effect by activating Bcl-2. At the same time, G-CSF can reduce the production of NO and prevent neurons from entering programmed cell death (Peña and Borlongan, 2015).

### Pro-inflammatory Cytokines

#### IL-1

IL-1 is synthesized and secreted by a variety of active cells, including glial cells and endothelial cells, and is a broad class of polypeptides. IL-1β is the main form in plasma, tissue fluids, and brain tissue. It not only synergizes with other cytokines to promote the activation of B lymphocytes and T lymphocytes, but also induces the production of other inflammatory mediators, strengthens the adhesion of leukocytes to endothelial cells, and regulates TNF-α and IL-6 (Diaz-Cañestro et al., 2019). CIRI can induce IL-1β production, upregulate ICAM-1expression, promote the adhesion of leukocytes to vascular endothelial cells, increase the adhesion of neutrophils to endothelial cells, and cause leukocytes to accumulate in the ischemic area and promote inflammation (Pradillo et al., 2017). This reaction aggravates cerebral ischemic damage. *in vitro* experiments have shown that IL-1β protects neurons under physiological conditions, but excessive IL-1β can damage neurons and other tissues. Anti-IL-1β treatment can alleviate brain edema and reduce infarct size after cerebral ischemia (Dziedzic, 2015), and activated protein-1 (AP-1) transcription factor JunD blunts ischemia/reperfusion-induced brain injury *via* suppression of IL-1β (Diaz-Cañestro et al., 2019).

#### IFN-γ

IFN-γ is a pro-inflammatory factor not expressed in normal brain tissue. After cerebral ischemic injury, T cells and NK cells are activated, releasing IFN-γ, and enhancing the ischemia-induced neurotoxicity. Studies have confirmed that IFN-γ is involved in the infiltration of microglia and inflammatory cells in cerebral ischemia (Li K. et al., 2017), and that IFN-γ also stimulates the production of interferon regulatory factor-1, induces the expression of NOS mRNA, and produces neurotoxic effects (Yilmaz et al., 2006).

#### IL-8

IL-8 has chemotactic activity and activates neutrophils in the early inflammatory response of nerve tissue, produces oxidative metabolites, releasing intracellular enzymes, and promotes inflammatory responses of nervous tissues in cerebral ischemia (Connell et al., 2015). The deleterious effects of IL-8 and its receptors in ischemic brain injury have been confirmed, and blocking IL-8 and its receptors alleviates brain damage. Serum CXCL8 (C-X-C Motif Chemokine Ligand 8, alias of IL-8) level is associated with the infarct volume and functional outcomes in patients with ischemic stroke (He et al., 2018). In a MCAO model, the application of the IL-8 receptor inhibitor repertaxin significantly reduced the infiltration of neutrophils into the brain parenchyma, reducing local inflammation and infarct volume (Villa et al., 2007).

#### IL-16

IL-16 expression is upregulated after cerebral ischemia, causing a pro-inflammatory response, and is closely related to the anatomical location of cell death.IL-16 is a key inflammatory cytokine in ischemic injury, and in addition to its inflammatory and chemotactic effects, it induces further IL-16 expression through fibrin deposition (i.e., the initial thrombotic process). IL-16 can induce the expression of TNF-α and IL-1β, both of which can increase vascular permeability and impair capillary integrity. IL-16 may also cause blood-brain barrier collapse and lead to edema formation and subsequent secondary damage (Chen A. et al., 2016).

#### IL-17

IL-17 induces the secretion of IL-6, IL-8, monocyte chemotactic protein-1, prostaglandin E2, and G-CSF from epithelial cells, keratinocytes, endothelial cells, and fibroblasts, and can upregulate the expression of ICAM-1 by these cells (Waisman et al., 2015). Upregulation of IL-17 mRNA can induce the expression of IL-1, IL-6, IL-8, TNF-α, and ICAM-1, which may aggravate the secondary inflammatory reaction after cerebral ischemia. If IL-17 is effectively blocked, the production of cytokines and brain tissue damage after ischemia are significantly reduced (Ma et al., 2018). The NOD-like receptor protein 3 (NLRP3) inflammasome is reported to exacerbate the injuring effect of the IL-23/IL-17 axis, then aggrevating CIRI (Wang H. et al., 2019). Others report that administration of rIL-17A nullifies the non-invasive vagus nerve stimulation (nVNS) induced M2 type of microglial polarization, which is an important mechanism underlying the nVNS-mediated neuroprotection against CIRI (Zhao X. P. et al., 2019).

#### IL-18

IL-18 is the most potent proinflammatory factor found in inflammatory cytokines so far. It plays an important role in regulating the immune and inflammatory responses of the body, and is involved in the occurrence, development, and rupture of atherosclerotic plaques. At present, little research on the relationship between IL-18 and cerebral ischemia has been conducted, but it is considered to be a pro-inflammatory cytokine similar in structure to IL-1β. Studies have shown that IL-18 mRNA is detected by reverse PCR at 48 h after cerebral ischemia, reaching a peak at 7–14 d. The peak expression of IL-1β mRNA was at 16 h, and it was then downregulated, suggesting that IL-18 may have a certain degree of regulation on the inflammatory response in the late stage of cerebral ischemia (Zhang et al., 2010).

#### ICAM-1

ICAM-1 is an important intercellular adhesion molecule involved in the adhesion of leukocytes to vascular endothelial cells. After cerebral ischemia-reperfusion, various cytokines such as IL-1, IFN-γ, TNF-α, and endotoxins can upregulate the expression of ICAM-1 and promote the strong adhesion to, and activation of, lymphocytes, neutrophils, eosinophils, and monocytes to endothelial cells (Li W. et al., 2017). Subsequently, a large number of plasma components (especially fibrinogen) accumulate in the blood vessel wall, resulting in local microcirculation blood flow dysfunction. Simultaneously, inflammatory cells that accumulate in the ischemic area release large amounts of toxic oxygen free radicals, proteolytic enzymes, and related molecules, thus increasing vascular permeability, destroying the blood-brain barrier, and causing toxic damage to neurons (Yang et al., 2018). Dexmedetomidine alleviates ischemia-reperfusion damage to rat brains and inhibits NF-κB and ICAM-1 expression in brain tissues (Li and Liu, 2017).

#### Leukotrienes (LTs)

LTs are divided into two categories: LTB4, with strong chemotactic action, and cysteine leukotrienes (cysLTs), including LTC4, LTD4, and LTE4. CysLTs are potent pro-inflammatory and immune modulating lipid mediators involved in inflammatory diseases and are boosted in human brains after an acute phase of cerebral ischemia (Bevan et al., 2008). LTC4, LTD4, and LTE4 are produced during cerebral ischemia in patients and experimental animals, which causes cerebral vasospasm and increases vascular permeability, leading to cerebral edema and cerebral ischemic injury. In an MACO model, LTC4 was perfused into a the internal carotid artery, and after 48–72 h, γ-glutamyl transpeptidase was reduced in brain capillaries, and blood-brain barrier permeability was increased (Garcia et al., 2017). CysLTs receptor antagonists have also been shown to reduce the expression of cysLTs induced by cerebral ischemia, and inhibit brain-blood barrier disruption, alleviate cerebral vascular permeability, and reduce cerebral edema (Shi et al., 2012; Yagami et al., 2013; Saad et al., 2015). Montelukast, a CysLTR1 antagonist has protective effects on acute, subacute, and chronic stages of brain injury in whole or focal cerebral ischemia rat models by reducing oxidative stress, inflammatory and apoptotic markers. Furthermore, it reduces glutamate and lactate dehydrogenase activity as well as infarct size elevated by CIRI (Saad et al., 2015). HAMI 3379, a selective CysLTR2 antagonist, has also been proven to protect against acute brain injury after focal cerebral ischemia in rats (Shi et al., 2012).

#### Monocyte Chemotactic Protein 1 (MCP-1)

Normal brain tissue barely expresses MCP-1, but vascular endothelial cells, astrocytes, activated leukocytes, and microglia can express MCP-1 in the case of cerebral ischemia. *in vivo* and *in vitro* experiments have confirmed that MCP-1 has chemotactic activity in monocytes, activates monocytes and macrophages, increases intracellular Ca^2+^ concentration, stimulates the production and release of superoxide anions, and releases lysozyme. It is also involved in cerebral ischemic injury by the chemotaxis of mononuclear macrophages infiltrating brain parenchyma and by upregulating the expression of macrophage adhesion molecules, such as the integrin family β2 and α4 molecules, and the production of cytokines IL-1 and IL-6 (Strecker et al., 2013). The mean cerebral infarction volume of MCP-1-knockout mice was 29% smaller than that of wild-type mice after 24 h in an MCAO model, and MCP-1 knockout mice had less expression of inflammatory cytokines and less brain damage after 6 h. This suggests that inhibition of MCP-1 signaling may be a novel therapeutic strategy to limit the volume of cerebral infarction after stroke (Hughes et al., 2002). Increased expression of MCP-1 is associated with monocyte accumulation and activation of glial cells in ischemic and hypoxic brain tissue, while activated glial cells produce a large number of neurotoxic factors, leading to neuronal degeneration and necrosis (Yuan et al., 2017).

### Cytokines With Dual Pro-inflammatory and Anti-inflammatory Effects

#### IL-6

IL-6 is a neurotrophic factor that is involved in inflammatory regulation and the immune response. The main cells producing IL-6 in brain tissue are astrocytes and microglia, which can be induced to produce IL-6 by stimulation and virus infection. IL-6 mRNA is mainly derived from neurons and is expressed in the ischemic area, cerebral cortex and hippocampus, and is most obvious in the ischemic peripheral area. The expression is highest after 24 h of reperfusion, and remains high in the later stages of CIRI (Fujita et al., 2009). The protective effect of IL-6 on cerebral ischemic injury may be related to the following factors: (1) inhibition of the production of IL-1 and TNF- α as it stimulates the production of circulating antagonists, such as soluble TNF receptors and IL-1 receptor antagonists, through negative feedback mechanisms; (2) induction of the expression of adrenocorticotropic hormone and hydrocortisone, promoting the expression of acute phase proteins, which have anti-protease and oxygen scavenging effects; and (3) exhibiting endogenous neuroprotection against N-methyl-D-aspartate receptor mediated damage (Fujita et al., 2009). However, studies have also shown that IL-6 is involved in the inflammatory response during cerebral ischemia. The detection of IL-6 in patients with acute cerebral infarction found that IL-6 in the cerebrospinal fluid and serum is an independent predictor of cerebral infarction: the greater the volume of cerebral infarction, the higher the level of IL-6, and this was not related to the location of the cerebral infarction (Wytrykowska et al., 2016). IL-6 increases vascular endothelial cell monolayer permeability *in vitro*, and contributes to ischemia-related blood-brain barrier dysfunction. One study showed that inhibiting the effects of IL-6 protein with systemic infusions of neutralizing antibodies attenuates ischemia-related increases in blood-brain barrier permeability by inhibiting IL-6, and modulates tight junction proteins after ischemia (Zhang et al., 2015b).

#### TNF-α

TNF-α is a pleiotropic cytokine that can cause leukocyte infiltration and tissue damage after cerebral ischemia. TNF-α causes damage to ischemic brain tissue: (1) by damage to vascular endothelial cells, changing their permeability; (2) through a variety of mechanisms increasing the adhesion of white blood cells and vascular endothelial cells, such as the upregulation of ICAM-1 and CD11/CD18; and (3) by interaction with endothelial cells leading to vascular dysfunction and induction of coagulation processes (Esposito and Cuzzocrea, 2009). IL-16 can upregulate the action of TNF-α, further aggravating the inflammatory reaction of the tissue, leading to further stenosis of the local vessels. TNF-α mechanistically contributes to cerebral edema by increasing blood-brain barrier permeability and is an underlying factor in the development of cerebrovascular abnormalities associated with preeclampsia complicated by placental ischemia (Warrington et al., 2015). However, it is also reported that TNF-α plays a protective role in ischemic brain tissue. TNF-α preconditioning can attenuate oxidative stress injury, inflammatory activity, and apoptosis level in ischemia/reperfusion-induced cerebral injury (Xu G. et al., 2017). Additionally, one study showed that cortical infarction and behavioral deficits are significantly exacerbated in TNF-knockout mice compared with wild-type mice. BM-chimeric TNF-knockout mice grafted with wild-type BM cells developed larger infarcts than BM-chimeric wild-type mice grafted with TNF-knockout BM cells, providing evidence that the neuroprotective effect of TNF is attributable to microglial-derived TNF (Lambertsen et al., 2009). Therefore, the relationship between TNF- α and ischemic stroke needs to be further explored.

## Anti-cerebral Ischemic Drugs Targeting Inflammatory Cytokines

The IL-8 receptor inhibitor repertaxin can inhibit neutrophil infiltration and reduce infarct volume in transient and permanent MCAO rat models. Investigating the effects of repertaxin on neurological function and inflammation in different treatment regimens and treatment windows revealed that the volume of cerebral infarction was significantly reduced at 24 h after CIRI, but there was no significant difference after 24 h (Villa et al., 2007). Kineret (Greenhalgh et al., 2010) is a recombinant human IL-1 receptor antagonist (rhIL-1ra) approved by the US FDA and the European EMA for the treatment of rheumatoid arthritis. In the MCAO model, IL-1ra can reduce infarct volume by 33% and edema by 57%. It penetrates the damaged blood-brain barrier, and immunofluorescence confirmed that it binds with IgG to rescue dying neurons. In a rat model of CIRI, it was found that an intravenous injection of the ICAM-1 antibody 1A29 (1 mg/kg) before ischemia or at 4 h after reperfusion could inhibit the aggregation of leukocytes, reduce the infiltration of inflammatory cells, and clearly reduce brain damage (Cao et al., 2009). The application of the block copolymer PEG-b- (PELG-g-PLL) as a potential TNF-α nanocarrier with sustained release significantly enhanced the bioavailability of TNF-α (Xu G. et al., 2017). The block copolymer PEG-b- (PELG-g-PLL) may therefore function as a potent nanocarrier for augmenting BI/RI pharmacotherapy, with unprecedented clinical benefits (Xu G. et al., 2017).

## Conclusions

A large number of experiments have confirmed that antioxidants can exert neuroprotective effects by scavenging free radicals and inhibiting lipid peroxidation. Antioxidant therapy can reduce cerebral ischemic injury to a certain extent, but the clinical effect is not ideal. In view of the complex mechanisms of inflammation in CIRI, especially its cascade reaction, there is no single control of a certain reaction or inhibition of a certain molecule that can achieve control of neuroinflammation. We believe that effective drug treatment studies can be focus on two aspects: the development of a free radical scavenger with multiple mechanisms of action, and the idea that free radical scavengers work better when combined with anti-inflammatory drugs. The development of a suitable method for combination therapy has become a hot topic.

## Author Contributions

LW wrote the initial draft. XW contributed to collecting literature. Figures and submission prepared by YY and ZJ. ZZ and XX prepared the final version. LG recommended a structure for the review, substantially advanced the draft.

## Conflict of Interest

The authors declare that the research was conducted in the absence of any commercial or financial relationships that could be construed as a potential conflict of interest.
